# The burden, risk factors and prevention strategies for drowning in Türkiye: a systematic literature review

**DOI:** 10.1186/s12889-024-18032-9

**Published:** 2024-02-20

**Authors:** Ali Işın, Amy E. Peden

**Affiliations:** 1 Department of Coaching Education, Faculty of Sports Sciences, Akdeniz University, 07070 Antalya, Türkiye; 2 School of Population Health, UNSW Sydney, Kensington, NSW 2052 Australia; 3 College of Public Health, Medical and Veterinary Sciences, James Cook University, Townsville, QLD 4811 Australia

**Keywords:** Injury, Injury Prevention, Public Health, Water Safety

## Abstract

**Introduction:**

Drowning is a public health problem in Türkiye, as in the rest of the world. This study aims to systematically review the literature on drowning in Türkiye with a focus on data sources, epidemiology, risk factors and prevention strategies. Methods: Literature searches were conducted using PubMed, SPORTSDiscus, Scopus, Web of Science, Turk MEDLINE, Google Scholar and Google Akademik (Turkish language). Studies (limited to original research written in English and Turkish) reporting drowning (unintentional and intentional; fatal and non-fatal) of residents and tourists in Türkiye were independently dual screened at the title and abstract and full text stages. Study quality was assessed using JBI checklists and evidence level assessed based on study design. Results: From a total of 917 studies, 49 met the inclusion criteria. Most (51%) focused on unintentional fatal drowning. Included studies were most commonly analytical cross-sectional studies (*n* = 23) and case series (*n* = 20) meaning the evidence level was low or very low for 48 (98%) studies. Fifteen studies examined drowning at the national level, while sub-national studies (*n* = 30) focused on urban areas across three provinces: Antalya (*n* = 6), Istanbul (*n* = 6), Izmir (*n* = 4). There was little consensus on risk factors beyond male drowning risk, and no data reported on implemented or evaluated drowning prevention interventions. Discussion: There is a need for more national-level studies to identify the causes of drowning and to guide intervention implementation and evaluation to inform policy makers and donors. Currently official data is limited in its detail, providing age and gender data only, hampering efforts to identify, and thus address, causal factors for drowning. Practical applications: There is currently very little evidence to inform investment in effective drowning prevention interventions in Türkiye. To improve this, data collection systems on drowning in Türkiye need to be strengthened via the development a national drowning registry.

**Trial Registration:**

#CRD42022382615.

**Supplementary Information:**

The online version contains supplementary material available at 10.1186/s12889-024-18032-9.

## Introduction

Drowning is recognised as a serious public health problem worldwide. In 2019, more than 230,000 people died due to drowning, mostly in low- and middle-income countries, making drowning the third leading cause of unintentional injury death globally (accounting for 7% of all injury-related deaths) [1]. Studies from several countries identify that such figures likely underreport the true burden of drowning due to the exclusion of water transport and disaster-related drowning [2–4], as well as intentional drowning [5]. Drowning can occur in any type of water, such as rivers, lakes, oceans, pools, bathtubs or buckets, and can be classified as fatal or non-fatal depending on whether the outcome of the initial drowning incident [6].

Türkiye, a Eurasian country with 783,577 km2 of land, is surrounded by four seas (the Mediterranean, the Aegean, the Black Sea, and the Marmara) and has many lakes, streams and rivers [7]. The country’s total coastline is 8,592 km long and the area of the coastal provinces accounts for 30% of the whole country. Türkiye’s most populous provinces are generally along the coast [8]. This gives more people access to the sea, thus increasing drowning risk. Moreover, with the rising temperatures in the summer months, more people participate in aquatic activities such as swimming, boating, etc. This leads to fatal and non-fatal drowning incidents in Türkiye [9]. Although there are lifeguards on all major beaches, people may choose to enter the water in more rural areas. Also, in rural areas, irrigation canals, lakes, dams, rivers and streams are seen as significant risk factors for drowning. It is thought that the number of drownings increases in these areas due to the lack of protective measures (such as warning signs and rescue equipment) [10].

Drowning is a significant issue across the European region [11], including in Türkiye, where the prevention of drowning is challenging due to a lack of reliable and comprehensive data on its burden and risk factors [7]. The number of drowning deaths and crude mortality rate in Türkiye is uncertain due to different data sources (media data, clinic reports and autopsy records) which use different definitions thus affect the accuracy of estimates of drowning mortality in Türkiye. Further, the exclusion of flood-related drowning deaths and water transportation-related drownings [10] also risks underrepresenting the true burden. Further, there is no total population level data capture on non-fatal drowning in Türkiye. Therefore, more comprehensive and consistent data on drowning in Türkiye are needed to inform prevention strategies and policies [7].

Given of the lack of consolidated information on drowning in Türkiye, this systematic literature review aimed to identify and synthesise the published literature on drowning burden, data sources, risk factors and prevention strategies in Türkiye, with the aim of informing next steps for drowning prevention in the country.

## Materials and methods

The protocol for this systematic review was prospectively registered with PROSPERO (#CRD42022382615) and conducted according to the Preferred Reporting Items for Systematic Reviews and Meta-Analysis (PRISMA) guidelines [12].

### Literature search

Searches were conducted using PubMed, SPORTSDiscus, Scopus, Web of Science, and Turk MEDLINE from inception to 9th December 2022. The inclusion and exclusion criteria are presented in Table 1. Search terms included drown*, immers*, submers*, swim*, a variety of aquatic locations (i.e., river, lake, sea, beach, pool) and Turk*. Full search strategies can be found in Table S1. These were tailored to suit each journal and based on consultations with a specialist librarian and a previous literature review of drowning [13]. Search strings were also devised in such a way as to capture more relevant information, for example, cases classified as drowning not just deaths or incidents in water, and swimming as it pertains to drowning prevention and not competitive swimming or the biomechanics of swimming.

**Table 1:** Inclusion and exclusion criteria for systematic review of the literature on drowning in Türkiye

Inclusion criteria	Exclusion criteria
· Peer-review literature	· Non-peer-reviewed
· Original research	· Non-original research (i.e., literature reviews, opinion pieces, editorials)
· Written in English or Turkish Language	· Non-English and Turkish language
· Limited to humans	· Non-human
· Case reports included if reports ≥ 5 cases	· Studies reporting < 5 cases
· Residents and tourists in Türkiye	· Turkish residents drowning outside of Türkiye
· Intentional and unintentional drowning	
· Fatal and non-fatal drowning	

After database searches were run, additional searches of boğulma* AND Türk were run using Google Akademik (Turkish language by author AI) and drown* AND Turk* in Google Scholar (English language by author AP) to identify any articles not found via database searches. Authors screened results until 10 pages of nil results. As a result of these searches, no new articles were identified. Databases were chosen based on their relevance to drowning from a previous review of drowning in a neighbouring region [13], in addition to the use of Turk MEDLINE and Google Akademik to capture Turkish literature not indexed in the other databases.

### Study selection

Two authors (AI and AP) conducted a dual independent review of the title and abstract followed by full-text screening with conflicts resolved via consensus. One Turkish-speaking author (AI) reviewed Turkish language literature, clarifying any concerns with author (AP). Study screening was performed using Covidence literature screening software [14].

### Data extraction

Data extraction was undertaken by one author (AI) with an independent quality check of 20% of included records undertaken by a second author (AP). Data extraction was undertaken using a Microsoft Excel Spreadsheet custom-built for this purpose. Data were extracted on the following aspects: Study characteristics (which included author name, year published, years of study, study population, study design and data source(s)), epidemiology, risk factors, and prevention strategies.

The epidemiology of drowning was reported as numbers, proportions, or rates per 100,000 for each population reported (overall, by age group, by year, by gender, etc.) in the included studies. No inferred rates were calculated. Drowning was described by outcome (fatal, non-fatal, both, not specified), and intent (unintentional, intentional, both, not specified) and examined at a total population level, as well as by age group and gender.

We coded the free text description of data sources, risk/protective factors, and prevention strategies by consensus. Risk/protective factors were those that had a significant association with the risk of drowning or drowning outcome (e.g., chi square tests of significance [*p* < 0.05], odds ratio, relative risk). We extracted prevention strategies that were proposed, implemented and/or evaluated. We classified prevention strategies as being primary (before the drowning occurs), secondary (reduce the impact of a drowning which has already occurred), or tertiary prevention (reduce the ongoing effects of a drowning incident) [15] and also aligned strategies to the Hierarchy of Control [16]. We also noted if the prevention strategy involved multi-sectoral action (as recommended by the WHO [17]) and which sectors were involved.

### Quality appraisal

Quality assessment of included studies was performed by two members of the review team using the Joanna Briggs Institute (JBI) Critical Appraisal Tools based on study type. The first author (X1) assessed all articles and then the other author (X2) randomly assessed 20% of the articles. Disagreements between the two authors were resolved by discussion. Checklists provide a score based on assessment of a range of study design criteria. Study design of the included studies were graded according to the National Health and Medical Research Council's (NHMRC) levels of evidence, which range from level I (a systematic review of Level II studies [randomised controlled trial]) to level IV (case studies with either post-test or pre-test/post-test outcomes) (Table S2).

## Results

**Fig. 1:**
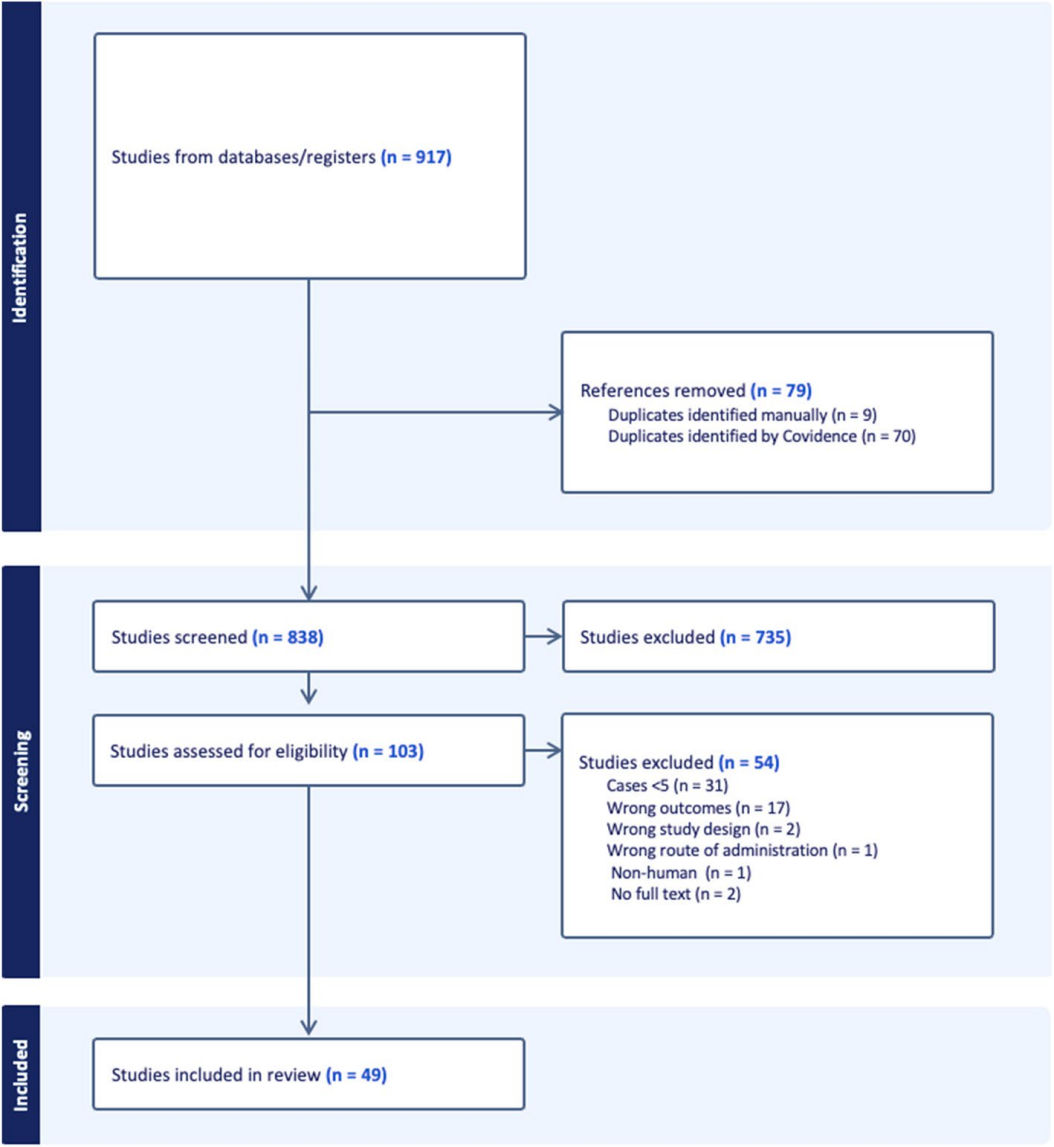
PRISMA flow chart

Database searching yielded 917 studies. After removal of 79 duplicates, 838 studies were screened by title and abstract for inclusion. Of these, 735 studies were deemed irrelevant and excluded. The remaining 103 full text studies were screened for eligibility. In total, 54 studies were removed at full text review and data were extracted from the remaining 49 studies which satisfied the inclusion criteria (Fig. 1).

### Study characteristics

Among the 49 included studies, the publication dates ranged from 2004 to 2022. The included studies were predominately analytical cross-sectional studies (*n* = 23; 47%) and case series (*n* = 20; 41%). The remaining studies comprised four prevalence studies, one qualitative study and one quasi-experimental study. Included studies mostly used autopsy data (*n* = 21; 43%) or medical reports (*n* = 13; 27%), followed by media reports (*n* = 8; 16%). Based on study design, the overall level of evidence was low, with almost all studies (*n* = 48) ranked as low or very low on the NHMRC Levels of Evidence criteria (Table S2). When assessed using JBI checklists based on study type, 23 studies (47%) recorded a score of 7 or above.

**Fig. 2:**
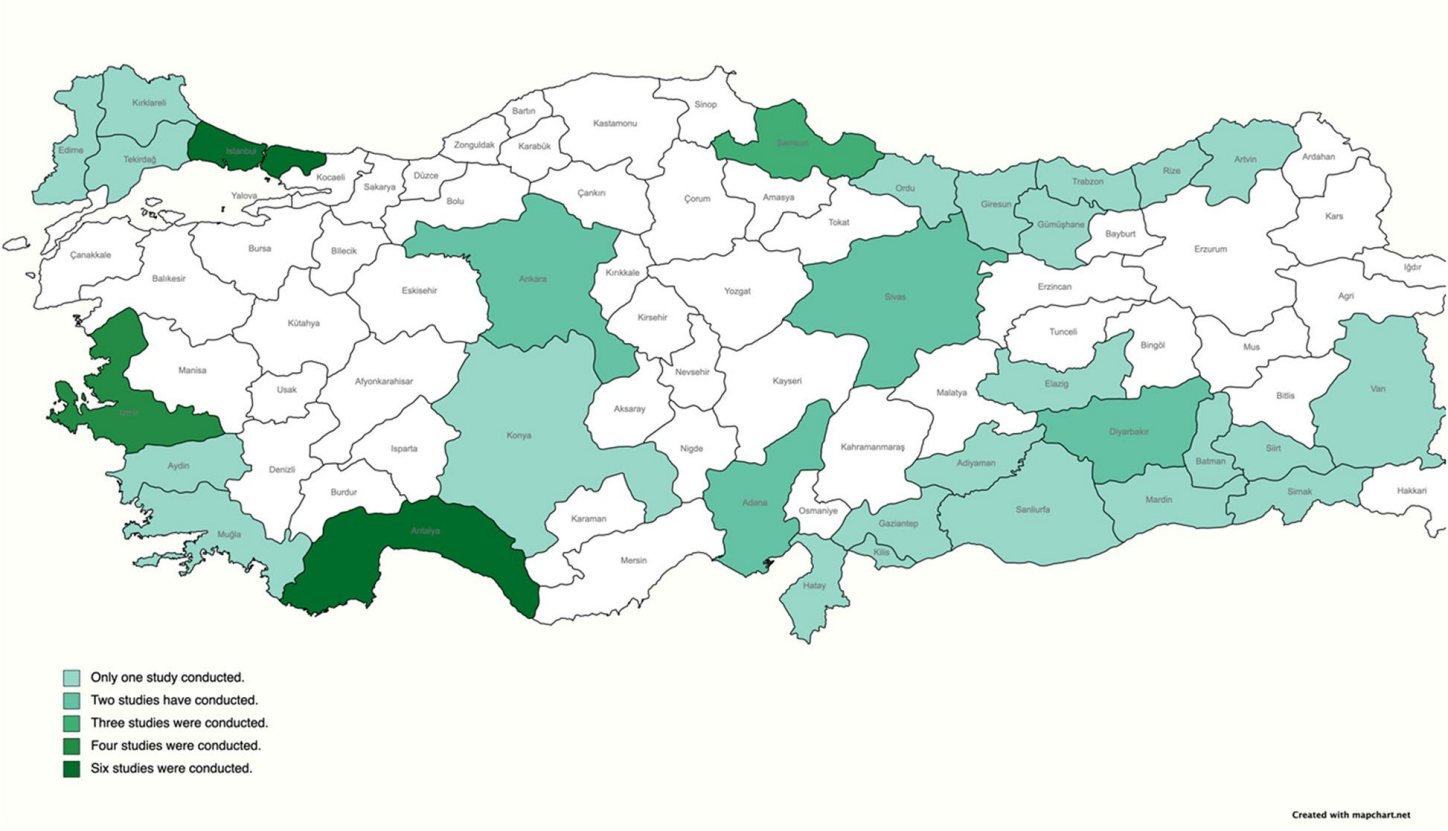
Heatmap of sub-national studies by location

Fifteen included studies reported drowning at the national level, while 16 studies reported drowning at the provincial level, most commonly in Antalya (*n* = 6), Istanbul (*n* = 6) and Izmir (*n* = 4) (Fig. 2). More than half of the included studies reported data at the sub-national level (*n* = 30; 61%), followed by 16 studies (33%) reporting national data and 3 studies (6%) reporting on foreign visitors to Türkiye. No studies examined drowning among migrants, either once they had arrived in Türkiye or while in transit. Most of the studies (27 out of 49; 55%) reported data from urban areas, while two studies (4%) reported data from rural areas. Some studies (20 out of 49; 41%) reported data from both urban and rural areas. Fatal drownings were the focus of 36 studies, while both fatal and non-fatal drownings were included in 12 studies. While 18 of these studies examined unintentional drownings only, seven examined both intentional and unintentional drownings. The remaining 24 studies did not distinguish between intentional and unintentional drownings (Table 2).

**Table 2:** Included studies (by location, time period, study population, data source, drowning outcome and intention)

Reference	Study location (name, national/sub-national; Rurality)			Time period	Study population	Data source	Outcome	Intention
Aşırdizer et al. 2005 [18]	Istanbul	S	U	1996–2020	Infant and adolescent—age ≤ 18	Autopsy reports	*F*	*U*
Atilgan et al. 2022 [19]	Antalya	S	U	2010–2019	All age	Autopsy reports	*F*	NS
Azmak (2006) [20]	Trakya region	S	B	1984–2004	All age	Autopsy reports	*F*	B
Barlas and Beji (2016) [21]	Istanbul	S	U	2007–2012	All age	Hazard event records	*F*	NS
Başol et al. (2012) [22]	Samsun	S	U	2005–2011	Age ≥ 18	Medical records	B	B
Beydilli et al. (2017) [23]	Antalya	S	R	2009–2014	Age ≥ 18	Medical records	*F*	*U*
Çakmakcı et al. (2021) [24]	Izmir	S	U	2008–2018	All age	Medical records	*F*	NS
Cantürk et al. (2009) [25]	Ankara	S	U	2003–2006	All age	Autopsy reports	*F*	NS
Cantürk et al.(2007) [26]	Istanbul	S	U	2000–2002	Children—age ≤ 18	Autopsy reports	*F*	NS
Çaylan et al. (2021) [27]	Türkiye	N	B	2014–2017	Children—Under 5	Death Notification System	*F*	B
Dirlik et al. (2015) [28]	Aydın	S	U	2002–2012	Children—age ≤ 18	Autopsy reports	*F*	NS
Dogan et al. (2010) [29]	Konya	S	U	2000–2007	All age	Autopsy reports	*F*	B
Esiyok et al. (2006) [30]	Türkiye	N	B	1992–2022	All age	Autopsy reports	*F*	NS
Güzel et al. (2013) [31]	Samsun	S	B	2005–2012	Children—age ≤ 18	Autopsy reports	B	NS
Hsieh et al. (2018) [32]	Türkiye	N	B	2012–2014	All age	WHO database	*F*	B
Işik and Eşitti (2015) [33]	Türkiye	N	B	2007–2012	All age	Media reports	B	NS
Işın et al. (2020) [7]	Türkiye	N	B	2005–2017	Children—age ≤ 18	Media reports	*F*	*U*
Işın and Peden (2022) [10]	Türkiye	N	B	2013–2019	All age	TurkStats and GBD data	*F*	*U*
Işın et al. (2021) [34]	Türkiye	N	B	2015–2019	All age—rescue-related drownings	Media reports	*F*	*U*
Ketenci et al. (2022) [35]	Eastern Black Sea Region	S	B	2009–2016	All age	Autopsy reports	*F*	*U*
Koca et al. (2019) [36]	Türkiye	N	B	2007–2013	All age—fatal diving accidents	Autopsy reports	*F*	*U*
Lakadamyalı et al. (2008) [37]	Alanya	N	R	2002–2006	All age	Autopsy reports	B	NS
Lapa et al. (2012) [38]	Türkiye	N	B	2006–2010	All age—recreational	Media reports	*F*	*U*
Lin et al. (2015) [39]	Türkiye	N	B	2006–2008	All age	WHO database	*F*	*U*
Mollaoğlu et al. (2013) [40]	Sivas	S	U	2009–2010	All age	Medical records	*F*	NS
Orhan (2020) [41]	Türkiye	N	B	2011–2016	All age—recreational activity	Media reports	*F*	*U*
Petrucci et al. (2019) [42]	Türkiye	N	B	1980–2018	All age—flood fatalities	EUFF database	*F*	NS
Şık et al. (2022) [43]	Izmir	S	U	2014–2020	Children—age ≤ 18	Medical records	B	*U*
Şık et al. (2021) [44]	Izmir	S	U	2009–2019	Children—age ≤ 18	Medical records	B	*U*
Şimşek and Satar (2013) [45]	Adana	S	U	2011–2012	All age	Medical records	B	NS
Söyüncü et al. (2008) [46]	Antalya	S	U	2001–2007	All age	Medical records	B	NS
Taşkesen et al. (2015) [47]	Southeast of Türkiye	S	B	2008–2013	Children—age < 15	Medical records	B	NS
Tunçez et al. (2022) [48]	Izmir	T	U	2015–2020	All age	Autopsy reports	*F*	B
Turgut (2012) [49]	Türkiye	N	B	2009	All age—multiple drowning syndromes	Media reports	*F*	*U*
Turgut and Turgut (2012) [50]	Türkiye	N	B	2005–2008	All age—rescue-related drownings	Media reports	*F*	*U*
Turgut and Turgut (2014) [9]	Türkiye	N	B	2007–2011	All age	Media reports	*F*	*U*
Turgut et al. (2016) [51]	Ankara-Antalya	S	B	NS	Secondary school students (10–14 yr)	Pre/Post tests of water safety knowledge in schools	NA	*U*
Uzun et al. (2009) [52]	Istanbul	T	U	1998–2002	All age	Autopsy reports	*F*	NS
Yayci et al. (2011) [53]	Istanbul	S	U	2001–2005	Children—age ≤ 18	Autopsy reports	*F*	*U*
Balcı et al. (2018) [54]	Muğla	S	U	2013–2016	Youth—age 15–24	Autopsy reports	*F*	NS
Yıldırım et al. (2020) [55]	Sivas	S	U	2008–2016	Children—age 0–6	Autopsy reports	*F*	NS
Küçük et al. (2020) [56]	Samsun	S	U	2010–2018	All age	Medical records	B	NS
Cömert et al. (2014) [57]	Istanbul	S	U	2007–2012	All age	Medical records	*F*	B
Türkoğlu et al. (2014) [58]	Elazığ	S	U	2005–2012	All age	Autopsy reports	*F*	NS
Beydili et al. (2016) [59]	Antalya	T	U	2012–2014	All age	Autopsy reports	*F*	NS
Demir et al. (2017) [60]	Van	S	U	2010–2014	Children—age ≤ 5	Autopsy reports	*F*	*U*
Tutanç et al. (2011) [61]	Diyarbakır	S	U	2002–2005	Children—age ≤ 18	Medical records	B	NS
Tutanç et al. (2011) [62]	Hatay	S	U	2003–2005	Children—age ≤ 18	Medical records	B	NS
Arslan et al. (2004) [63]	Adana	S	U	1997–2001	Children—age ≤ 18	Autopsy reports	*F*	NS

*N* National, *S* Sub-national, *T* Tourists, *U* Urban, *R* Rural, *B* Both. *F* Fatal, *NA* Not applicable, *U* Unintentional, *NS* Not Specified. *TurkStat* Turkish Statistical Institute, *GBD* Global Burden of Disease, *WHO* World Health Organization, *EUFF* European Flood Fatalities

^*^Medical records include clinical data, patient information form, medical charts, and nursing records

### Burden and risk factors

One of the more commonly reported risk factors for drowning in Türkiye was gender [7, 10, 27, 31, 34]. Three studies presented drowning mortality rates per 100,000 people [7, 9, 34]. In these studies, the drowning rates for males were 1.8 (between 2005 and 2017), 0.52 (2015–2019) and 1.44 (2007–2011), respectively, while the corresponding rates for females were 0.48, 0.06 and 0.28 (Table 3). Only one study reported a higher proportion of females drowning (60%) than males (40%), though case numbers were small [47].

**Table 3:** Numbers of fatal drowning by Gender

Reference	Study time period	Fatal—Drowning (n)		Fatal—Drowning (%)		Fatal—Drowning rate / 100,000 pop	
		**Male**	**Female**	**Male**	**Female**	**Male**	**Female**
Aşırdizer et al. (2005) [18]	1996–2000	7	4	63.6	36.4	-	-
Azmak (2006) [20]	1984–2004	37	4	90.2	9.8	-	-
Beydilli et al.(2017) [23]	2009–2014	116	39	74.8	25.2	-	-
Çakmakçı et al. (2021) [24]	2008–2018	70	47	59.8	40.2	-	-
Cantürk et al. (2009) [25]	2003–2006	88	23	79.3	20.7	-	-
Cantürk et al. (2007) [26]	2000–20002	61	18	77.2	22.8	-	-
Çaylan et al. (2021) [27]	2014–2017	148	75	66.4	33.6	-	-
Dirlik et al. (2015) [28]	2002–2012	33	6	84.6	15.4	-	-
Doğan et al. (2010) [29]	2000–2007	6	1	85.7	14.3	-	-
Güzel et al. (2013) [31]	2005–2012	42	13	76.4	23.6	-	-
Işın et al. (2020) [7]	2005–2017	2732	687	79.1	20.9	1.8	0.48
Işın and Peden (2022) [10]	2013–2019	3985	1019	79.6	20.4	-	-
Işın et al. (2021) [34]	2015–2019	213	24	89.9	10.1	0.52	0.06
Ketenci et al. (2022) [35]	2009–2016	36	5	87.8	12.2	-	-
Koca et al. (2019) [36]	2007–2013	49	3	94.0	6.0	-	-
Lakadamyali et al. (2008) [37]	2002–2006	24	8	75.0	25.0	-	-
Lapa et al. (2012) [38]	2006–2010	125	4	96.8	3.2	-	-
Orhan et al. (2020) [41]	2011–2016	200	36	84.7	15.3	-	-
Şık et al. (2021) [44]	2009–2019	62	27	69.7	30.3	-	-
Şimşek and Satar (2013) [45]	2011–2012	25	16	61.0	39.0	-	-
Söyüncü et al. (2008) [46]	2001–2007	23	11	67.6	32.4	-	-
Taşkesen et al. (2015) [47]	2008–2013	4	6	40.0	60.0	-	-
Turgut (2012) [49]	2009	25	6	80.6	19.4	-	-
Turgut and Turgut (2012) [50]	2005–2008	82	32	72.0	28.0	-	-
Turgut and Turgut (2014) [9]	2007–2011	2703	513	84.0	16.0	1.44	0.28
Uzun et al. (2009) [52]	1998–2002	8	2	80.0	20.0		
Yayci et al. (2011) [53]	2001–2005	142	25	85.0	15.0	-	-
Küçük et al. (2020) [56]	2010–2018	12	1	92.3	7.7	-	-
Türkoğlu et al. (2014) [58]	2005–2012	68	32	68.0	32.0	-	-
Beydili et al. (2016) [59]	2012–2014	69	20	77.5	22.5	-	-
Demir et al. (2017) [60]	2010–2014	23	20	53.5	46.5	-	-
Tutanç et al. (2011) [62]	2003–2005	10	2	83.3	16.7	-	-

Studies showed different rates in different age groups, with different data sources, and focusing on different regions. However, the general trend was that about 70% of drowning cases were male. Işın et al. (2020) reported that the drowning rate for children under 18 years of age was 1.18 per 100,000 for males and 0.48 for females. It also showed that the risk of fatal drowning was almost four times higher for males (relative risk: 3.98) than females [7].

Few and varied mortality rates were reported in the included studies because of differences in data sets and populations. Turgut and Turgut (2014) found that drowning rate of 0.89 per 100,000 people in Türkiye based on media reports [9]. A similar study by Işın et al. (2020) found a rate of 1.17 per 100,000 children aged 0–18 years [7]. Çaylan et al. [27] found that the rate in children under 5 years of age decreased from 1.1 per 100,000 population in 2014 to 0.7 in 2017. In another study also conducted in children [7], it was reported that the rate of drowning, which was in an upward trend from 2005 to 2010, decreased every year until 2017 to 0.78 per 100,000 children after peaking in 2010. In a study conducted on the whole population [10], it was found that the drowning rate has been on a downward trend every year since 2015 (1.24 per 100,000 people) and decreased to 0.64 in 2019 (Table 3).

Out of 16 studies that reported number of deaths in age groups, only 10 presented data for the 0–19 years age group. The total number of deaths reported varied from 1 to 1,086. There was no consensus on the age group with the highest burden of drowning; a population-based study showed the 65 + years age group as recording the highest number of drowning cases [10], while a population-based drowning study showed high drowning numbers in the 10–14 years age group [7] (Table 4). A study focusing on child drowning found that the drowning rate per 100,000 children by age group varied from a low of 0.73 for 0–4 year-olds, increasing with age to a high of 2.11 for adolescents aged 15–17 years [7]. According to a study of rescue-related drowning, the age group with the highest risk of drowning per 100 000 persons was 15–24 years (1.28), followed by 25–34 years with 0.78 [34].

**Table 4:** Numbers of fatal drowning by age group

	Age group													
**Reference**	0–4	5–9	10–14	15–19	20–24	25–29	30–34	35–39	40–44	45–49	50–54	55–59	60–64	65 +
Cantürk et al. (2019) [25]	42 (0–18 yr)				18 (19–28 yr)		20 (29–38 yr)		14 (39–48 yr)		9 (49–58 yr)		8 (59 yr +)	
Çaylan et al. (2021) [27]	25 (< 1 yr) 197 (1–5 yr)													
Dirlik et al. (2015) [28]	13	6	10	10 (15–18 yr)										
Güzel et al. (2013) [31]	7	19	29 (10–18 yr)											
Işın et al. (2020) [7]	591	688	1086	1055 (15–17 yr)										
Işın and Peden (2022) [10]	442	348	414	621	445	360	278	238	207	185	192	206	194	802
Işın et al. (2021) [34]	1	34 (5–14 yr)		83 (15–24 yr)		49(25–34 yr)		33 (35–44 yr)		22 (45–54 yr)		83 (55 yr +)		
Lapa et al. (2012) [38]	28 (0—18 yr)				64 (19—40 yr)				37 (41 yr +)					
Orhan (2020) [41]	23 (0–9 yr)		101 (10–19 yr)		50 (20–29 yr)		32 (30–39 yr)		18 (40–49 yr)		6 (50–59 yr)		3 (60 yr +)	
Şimşek and Satar (2013) [45]	4	19 (5–15 yr)		18 (15–65 yr)										
Taşkesen et al. (2015) [47]	8	2												
Yayci et al. (2011) [53]	25	32	36	74 (15–18 yr)										
Türkoğlu et al. (2014) [58]	21 (0–9 yr)		35 (10–19 yr)		17 (20–29 yr)		11 (30–39 yr)		4 (40–49 yr)		5 (50–59 yr)		7 (60 yr +)	
Beydili et al. (2016) [59]	41 (< 65 yr)													48
Demir et al. (2017) [60]	43 (< 5 yr)													
Arslan et al. (2004) [63]	8 (0–6 yr)	16 (7–11 yr)	20 (12–15 yr)	18 (16–18 yr)										

Fatal drownings by water location were reported in 16 studies. In Türkiye, the most common environments where fatal drownings occur were Beach/Sea, Stream/River/Creek, and Irrigation channel, respectively. The Beach/Sea was the most common drowning location in 5 studies, followed by Stream/River/Creek in 4 studies and lake in 2 studies. Bucket, Irrigation Channel, Hole/Well and Pool were each the most frequent drowning location in 1 study (Table 5). The sea/beach was the most common place for drowning across all age groups, but buckets were the main cause of drowning for children aged 0–4 years, while streams/rivers/creeks and irrigation channels were more prevalent for older children. Among rescuers, lakes/ponds and rivers were frequent drowning sites (Table 6).

**Table 5:** Fatal drownings by water location

Reference	Study time period	Study population	Fatal—Drowning (n)								
			**Beach/Sea**	**Pool**	**Stream/River/Creek**	**Lake/Pond**	**Irrigation Channel**	**Dam**	**Hole/Well**	**Bucket**	**Others**
Aşırdizer et al. 2005 [18]	1996–2000	Infant and adolescent—age ≤ 18								11	
Atilgan et al. 2022 [19]	2010–2019	All age	116	58							
Azmak (2006) [20]	1984–2004	All age	5		30			2	4		
Çakmakçı et al. (2021) [24]	2008–2018	All age	116	1							
Dirlik et al. (2021) [28]	2002–2012	Children—age ≤ 18	5	4	6	3	16				5
Esiyok et al. (2006) [30]	1992–2022	All age							21		7
Güzel et al. (2013) [31]	2005–2012	Children—age ≤ 18	41	6	2	2					4
Işın et al. (2020) [7]	2005–2017	Children—age ≤ 18	580	182	867	482	640	209	182		163
Işın et al. (2021) [34]	2015–2019	All age—rescue-related drownings	59	7	81	27	26	30	7		
Ketenci et al. (2022) [35]	2009–2016	All age	4		30	7					
Lapa et al. (2012) [38]	2006–2010	All age	61		21	35		12			
Mollaoğlu et al. (2013) [40]	2009–2010	All age		2	1						4
Turgut (2012) [49]	2009	All age—multiple drowning syndromes	7	2	14	5	2		1		
Turgut and Turgut (2012) [50]	2005–2008	All age—rescue-related drownings	20	5	37	41	6		3		1
Turgut and Turgut (2014) [9]	2007–2011	All age	856	382			405

**Table 6:** Risk factors identified in the included literature

Reference	Risk/Protective Factor	Specific detail	Measure of significance (i.e. relative risk, statistical significance etc.)
Çaylan et al. (2021) [27]	Gender	Male	Males significantly more likely to drown than females (*p* = 0.039)
	Area	Areas away from the home	Drowning more likely in areas away from the home as compared home or its close vicinity (*p* = 0.001)
	Season	Winter*	Seasonal differences in drowning with lower risk in Winter (*p* < 0.001)
Güzel et al. (2013) [31]	Gender	Male	The drowning rate was statistically higher in males (42 patients, 76.4%) than females (13 patients, 23.6%) (*p* < 0.001)
Işın et al. (2020) [7]	Gender	Male	Males were four-times more at risk (RR:3.98 CI: 3.66–4.32) than females
	Age	15–17 years	Children aged 15–17 years had the highest crude drowning rate (2.11 per 100,000 persons)
	Season	Summer	Compared to winter, the highest risk of drowning was in the summer (RR = 12.45)
Işın and Peden (2022) [10]	Gender	Male	Males significantly more likely to drown than females (*p* < 0.001)
	Age	65 + years	Aged 65 + years had the highest drowning rate (1.72 per 100,000 persons)
Işın et al. (2021) [34]	Season	Summer^a^ and Spring	Rescues more likely to be successful in Summer (*p* = 0.04) and less successful in Spring (*p* = 0.029)
	Activity	Swimming^a^ and non-water related recreation	Rescues more likely to be successful when victim swimming (*p* = 0.001) and more likely to be unsuccessful when having a non-water related recreation (*p* = 0.032)
	Location	Beach/sea^a^	Rescues more likely to be successful at beach/sea (*p* < 0.001)
	Gender	Female	Females were significantly more likely to fatally drown while conducting a bystander rescue while having a picnic (X^2^ = 6.333; *p* = 0.023)
	Gender	Male	Significantly higher risk of drowning while undertaking a bystander rescue for males
	Age	15–24-year-olds	15–24-year-olds (but most age groups compared under 5 s) (RR: 82.21, CI: 11.44–590.56)
Şık et al. (2021) [44]	Vital signs	Predictors of hospital admission	A Szpilman score of ≥ 4 [ (OR) = 12.109, 95% CI: 2.327–63.010, p: 0.003], a lactate level of > 2 mmol/L (OR = 4.390, 95% CI: 1.365–14.121, p: 0.013), and pathologic CXR findings (OR = 19.500, 95% CI: 3.761–101.112, *p* < 0.001) were identified as predictors of hospital admissions
	Receipt of CPR	Predictors of hospital admission	Rate of patients who received CPR was higher in the group admitted to the hospital (*p* < 0.001)
	Vital signs	Poorer outcomes	Evaluating the 8 patients with poor outcomes, they had lower body temperature (p: 0.015), Glasgow Coma Score (*p* < 0.001), pH (p: 0.012), and bicarbonate (p: 0.016) levels and higher Szpilman score (*p* < 0.001), AST (p: 0.009), ALT (p: 0.011), and lactate (p: 0.003) levels, with longer duration time of CPR (p: 0.03)
	NIV treatment	Shorter stay in hospital^a^	Total length of stay in the PICU and in the hospital was shorter in patients who underwent NIV treatment (p: 0.026, p: 0.001)

*RR* Relative risk, *CI* Confidence interval, *OR* Odds ratio, *CXR* Chest X-ray, *AST* Aspartate aminotransferase, *ALT* Alanine aminotransferase, *CPR* cardiopulmonary resuscitation, *NIV* Noninvasive ventilation

^a^Denotes protective factor

Beyond gender, age and water location, several other drowning risk and protective factors were identified in the included literature. Results differed with respect to season, with winter found to have statistically significant lower drowning risk than Summer [27], while in Summer drowning rescues were more likely to be successful when compared to other seasons [34]. Among fatal and non-fatal drowning of children < 18 years, receipt of CPR and Noninvasive ventilation (NIV) treatment were associated with survival to hospital admission and a shorter stay in hospital respectively, whereas poorer vital signs led to poorer outcomes [44] (Table 6).

### Prevention strategies

Identified prevention strategies included supervision for children aged ≤ 18 years, first aid education, data/research, rescue skills education (including throw rescues) and training, and swimming education. All were proposed strategies with no implementation or evaluation reported. All were classified as administrative on the Hierarchy of Control, representing lower order strategies in terms of likely effectiveness. Strategies were reasonably evenly spread across primary (four strategies), secondary (four strategies), and tertiary (three strategies) prevention. Most of the strategies (nine out of 11) involved more than one sector, with education and health being the most commonly co-occurring sectors (Table 7).

**Table 7:** Prevention strategies

Prevention strategy	Reference	Primary, secondary or tertiary prevention	Proposed, implemented or evaluated	Hierarchy of Control	Strategy span multiple sectors? (Y/N)	If yes, which sectors?
Supervision	Güzel et al. (2013) [31]	Primary	Proposed	Administrative	N	-
	Işın et al. (2020) [7]	Primary	Proposed	Administrative	Y	Education
First Aid Education	Güzel et al. (2013) [31]	Tertiary	Proposed	Administrative	Y	Health
	Işın et al. (2021) [34]	Tertiary	Proposed	Administrative	Y	Education, Health
	Ketenci et al. (2022) [35]	Tertiary	Proposed	Administrative	N	-
Data/research	Işın et al. (2020) [7]	Primary	Proposed	Administrative	Y	Health, Law Enforcement, Coastguard
	Işın et al. (2021) [34]	Primary	Proposed	Administrative	Y	Health, Law Enforcement, Coastguard
Rescue skills education and training	Işın et al. (2021) [34]	Secondary	Proposed	Administrative	Y	Education, Health
Swimming Education	Ketenci et al. (2022) [35]	Secondary	Proposed	Administrative	Y	Education
	Lapa et al. (2012) [38]	Secondary	Proposed	Administrative	Y	Education
	Turgut et al. (2016) [51]	Secondary	Proposed	Administrative	Y	Education

## Discussion

This study aimed to identify and synthesise the studies that have addressed drowning in Türkiye to date, examining data sources, epidemiology, risk factors and prevention strategies. Despite being a public health concern across Europe [11], our review identifies limited literature on drowning in Türkiye and low consensus on drowning risk factors. This lack of understanding on causal factors for drowning in Türkiye is thus manifest with no implementation or evaluation of drowning prevention strategies identified in the included literature [7, 64].

Little is known about the crude drowning rate in Türkiye. The most important reason for this is that most of the conducted studies in Türkiye were based on autopsy or clinical/medical reports. Studies using these sources are not generalisable as they usually focus on a single centre (hospital, forensic medicine) or a province/region. This was insufficient data presented in many of the included studies to calculate mortality rates. In addition, many population-based studies used media reports of drowning as their source of data. While media reporting can be useful in the absence of routinely collected data, and in Türkiye supplements the meagre detail provided from the national statistics authority [10], it is not without its limitations. Previous research has indicated a bias towards more newsworthy incidents and incidents which occurred in urban settings [65]. Therefore, such data must be interpreted with caution and provides further support for the establishment of detailed and timely routine data collection on drowning such as via a national registry [66].

Where drowning mortality rates were reported, the rates among children were lower than those of neighbouring countries such as Iran, albeit with different data capture methods used [13].

Studies presenting crude drowning rates of different years and populations in Türkiye showed that drowning in Türkiye has been on a decreasing trend recently. Declining drowning rates in Türkiye appear to mirror those reported globally [67], as greater effort and funding is directed toward the issue [68], particularly investment in those interventions known to be effective in young children [69]. However, there is a need to expand this investment into the adolescent age group who experience high drowning rates with relatively lower investment [70]. Additionally, there is a need to ensure drowning fatalities across both urban and rural settings are captured [10], as well as better exploration of the impact of non-fatal drowning, particularly on the Turkish health system.

There was little consensus on risk factors for drowning in Türkiye, within the identified literature, aside from the consensus regarding male drowning risk being greater than female [7, 10, 34]. This is broadly consistent with many other studies globally [67, 71–73].Based on the included studies, three possible reasons may account for the higher drowning rates among male in Türkiye; first, being males are more exposed to water than female. Thus, they spend more time in the water doing activities such as fishing, swimming, cooling off, boating, etc. [7, 9, 38]. Another reason could be that males are less likely to wear life jackets than female [7]. Finally, it is believed that male’s participation in the above activities under the influence of alcohol and drugs increases the risk of drowning in favour of male. Although the data didn’t meet the criteria to be included as a risk factor in our analysis, three studies suggested that alcohol consumption may be a preventable risk factor for drowning in Türkiye [37, 46, 59], particularly among males [37]. However, studies examining the impact of alcohol on drowning in Türkiye should consider the use of objective measures of alcohol consumption and intoxication such as recording blood alcohol concentration.

Another risk factor was age [7, 10, 27, 34]. Most of the included studies focused on children and adolescents, but some also evaluated all age groups. The results of these studies showed that children, adolescents, and individuals over 65 years of age had a higher risk of drowning than other ages. Effective drowning prevention interventions for young children are well understood, comprising active supervision, restricting access to water, water familiarisation [7] and cardiopulmonary resuscitation (CPR) as a tertiary response [44]. It may be that greater public education on strategies to reduce child drowning risk are needed within Türkiye [51], though the baseline knowledge is not currently known. The results of a study on drowning in swimming pools in Türkiye highlight the need to provide safer environments to prevent drowning in swimming pools. It is stated that lack of adequate safety measures and supervision is the cause of a significant proportion of child drownings. It was concluded that there is a need to close the pool edges with safety fences and to raise awareness of public by hanging information and warning signs at the edge of the pool [64], although such legislation is yet to have been implemented. While such approaches are likely to reduce drowning among young children, globally there is little evidence regarding effective drowning prevention interventions for adolescents and older people [70, 74].

Seasons were another risk factor for drownings in Türkiye. Fatal drowning cases increased in summer and decreased in winter [7, 27, 34]. People attended water environments for activities such as cooling off, swimming, fishing, boat trips, etc. [7]. Especially in the summer months, when the temperatures rose and the schools closed, people visited water environments such as sea, dam, lake, etc. more often [9, 64]. This led to more drownings due to a lack of supervision [7], inability to swim [64], swimming in areas without lifeguards [49], etc. Therefore, the authors recommended swimming education as a prevention strategy [51]. In addition, we recommend increased public education and awareness campaigns regarding drowning risk reduction strategies ahead of high-risk periods such as summer and school holidays.

Aquatic location was also identified as a risk factor for drowning, although there was little consensus in the included studies. Türkiye has many natural water bodies including four seas, numerous lakes, dams, and rivers [7], leading to natural water bodies being a leading location for all-age drowning. Results of this review show that drownings in areas close to the coastline were mostly in the sea [19, 24, 31], while lake, rivers and irrigation canals were the main drowning places in landlocked or inland areas [58]. the findings of the current systematic review showed that most studies focused on drownings in a single province or region. This resulted in different locations being the leading sites for drowning cases/deaths for children and adults, based on their geographical location. Drowning prevention interventions in Türkiye must be tailored to the accessible water bodies and practices around interaction with water in the different localities.

Although still facing drowning risk from natural waterbodies where adults drown, such as the sea and rivers, creeks and streams, this review also highlight the drowning risk for children posed by buckets [18] and irrigation channels [7, 28]. Updated research is needed to determine whether water storage practices have changed over time since Asurdizer et al.’s analysis of cases between 1996 and 2000 [18], including the potential role of water and sanitation hygiene advancements in changing child drowning risk profile. An absence of adult supervision combined with a lack of swimming ability contribute to drowning risk in irrigation channels. Therefore, parental education campaigns on supervision, as well as the provision of basic swimming and water safety education at the primary school level in Türkiye may assist in preventing future drowning incidents [50].

With respect to the ocean, Işın et al. [34] analysed the drownings of rescuers and found that rescues were more successful in the sea. The main reason for this is that seas are places where lifeguards are present and are visited by more people than other water environments. This increases the chance of rescue when more professionals intervene to save drowning people. Therefore, Işın et al. [34] suggested that rescue skill training and education would be an important prevention strategy, especially to prevent multiple drowning deaths.

Finally, lack of official data and limited data are considered as a barrier to the calculation of the burden of drowning in Türkiye [7]. Failure or limited determination of the burden of drowning and its underlying causes delays the planning of drowning prevention strategies. Previous studies in Türkiye have reported inadequate official records on drowning [7, 9, 34, 64]. Due to the limited availability of official sources, most studies on drowning in Türkiye have obtained data either from autopsy reports [19, 59] or medical reports (patient information form, electronic medical records, medical charts, and nursing records, etc.) [24, 43, 44]. However, such studies investigated drowning by analysing patient records or autopsy data from a region, a province, or one or more hospitals. Therefore, these studies were not successful in providing generalisable data on the total population burden of drowning in Türkiye due to their relatively small samples. Due to the lack of official records or limited information available to analyse the burden of drowning, researchers have analysed drowning in Türkiye from cases obtained from media reports [7, 9, 34, 64]. Although this type of research has some reported limitations, it has provided important findings because of its generalisable conclusions and its contribution to revealing the main gaps in Türkiye to prevent drowning. Official mortality data, triangulated with police and media reports, are needed to identify causal factors to inform, and in future evaluate, risk reduction initiatives. Although Işın and Peden (2022) obtained data from TurkSTAT, which use the death notification system, only gender and age group were analysed in the study because TurkSTAT provides very limited information [10]. While this contributes to the epidemiology of drowning in Türkiye, it is insufficient to formulate prevention strategies. As has been proposed in other European countries, a National Drowning Registry needs to be developed in Türkiye in order to collect drowning data efficiently [66]. The adoption of a non-fatal drowning definition that is consistently applied to capture non-fatal drowning cases in Türkiye in this registry would also be advisable.

### Strengths and limitations

To the best of our knowledge, this is the first systematic review of the published literature on drowning in Türkiye in terms of data sources, epidemiology, risk factors and prevention strategies. It is bolstered by examining publications in both English and Turkish language, as well as exploring publications from inception. However, it is not without its limitations. Turkish language studies could only be screened by one author due to the native language of the second author, which may have weakened the rigour around article screening and data extraction. Search strategies using different terms or combinations of terms, may have produced different results in terms of literature yielded. This review included primary studies published in peer-reviewed literature only. There may also be relevant information on the issue of drowning and its prevention within Türkiye published in the grey literature. The heterogenous nature of the studies made comparison difficult and a meta-analysis not possible.

## Conclusion

This research has highlighted the need for more generalised studies to better understand and estimate the burden of drowning deaths in Türkiye. Most of the studies were autopsy-based and focused on specific regions, or cities, which limited their generalisability. Thus, the burden of drowning in Türkiye was mostly calculated with media reports, which had some limitations and biases. There is a need for more research to support greater consensus on risk factors, to inform prevention interventions. However, the lack of accurate and comprehensive data remains a significant barrier to advancing drowning prevention efforts in Türkiye. We recommend the establishment of a national drowning data registry to capture fatal drowning incidents, before considering the inclusion of non-fatal drowning events. The consistent collection and timely analysis of such data are vital to saving lives from drowning in Türkiye.

### Supplementary Information

**Supplementary material 1. **

**Supplementary material 2. **

## Data Availability

All data generated or analysed during this study are included in this published article (and its Supplementary information files).
