# Supplementary material for: The burden, risk factors and prevention strategies for drowning in Türkiye: a systematic literature review
Source: BMC Public Health. 2024 Feb 20;24:528. doi: 10.1186/s12889-024-18032-9 (PMC10877921; doi:10.1186/s12889-024-18032-9)
Supplement: Supplementary file 1 — Supplementary material 1. [file 12889_2024_18032_MOESM1_ESM.docx]

**Table S1. Search Strategy**

| **Search number** | **Search strategy** | **Result number** |
| --- | --- | --- |
| **PubMed** | | |
| 1 | drown* | 7,322 |
| 2 | (immers*) OR (submers*) | 54,224 |
| 3 | Injur* | 1,386,710 |
| 4 | (#2) AND (#3) | 2,235 |
| 5 | (swim*) AND (education) | 4,192 |
| 6 | ((#1) OR (#4) OR (#5) | 13,300 |
| 7 | ((((((river) OR (lake)) OR (sea)) OR (beach)) OR (water safety)) OR (pool) | 529,042 |
| 8 | (#7) AND (drown*) | 1,117 |
| 9 | (#6) OR (#8) | 13,300 |
| 10 | (#9) AND (Turk*) | 182 |
| **Web of Science** | | |
| 1 | (((ALL=(immers* OR submers* AND injur*)) OR ALL=(swim* AND education)) OR ALL=(river OR lake OR sea OR beach OR pool OR water safety)) AND ALL=(drown* AND Turk*) | 100 |
| **SCOPUS** | | |
| 1 | ((immers* OR submers*) AND injury) OR (swim* and education) AND (drown*) AND Turk* | 265 |
| **SportsDiscus** | | |
| 1 | immers* OR submers* AND injur* OR swim* AND education AND drown* AND Turk* | 284 |
| **Turk MedLINE** | | |
| 1 | drown* | 86 |
| **Google Scholar** | | |
| 1 | drown* AND Turk* | 0 additional studies |
| **Google Akademik** | | |
| 1 | boğulma* AND Türk* | 0 additional studies |
