# Supplementary material for: The burden, risk factors and prevention strategies for drowning in Türkiye: a systematic literature review
Source: BMC Public Health. 2024 Feb 20;24:528. doi: 10.1186/s12889-024-18032-9 (PMC10877921; doi:10.1186/s12889-024-18032-9)
Supplement: Supplementary file 2 — Supplementary material 2. [file 12889_2024_18032_MOESM2_ESM.docx]

**Table S2.** JBI Checklist Assessment and [Australian] National Health and Medical Research Council (NHMRC) Level of Evidence for Included Articles (N=49)

| **Author name** | **JBI Checklist Type** | **Score** | **Overall appraisal** | **Level of Evidence (NHMRC)** |
| --- | --- | --- | --- | --- |
| Aşırdizer et al. 2005 [43] | Analytical cross-sectional studies | 6 | Include | IV |
| Atilgan et al. 2022 [40] | Case series | 8 | Include | IV |
| Azmak (2006) [47] | Case series | 7 | Include | IV |
| Barlas and Beji (2016) [48] | Analytical cross-sectional studies | 4 | Include | IV |
| Başol et al. (2012) [49] | Case series | 7 | Include | IV |
| Beydilli et al. (2017) [50] | Analytical cross-sectional studies | 6 | Include | IV |
| Çakmakcı et al. (2021) [41] | Case series | 9 | Include | IV |
| Cantürk et al. (2009) [51] | Analytical cross-sectional studies | 5 | Include | IV |
| Cantürk et al.(200**7**) [52] | Analytical cross-sectional studies | 6 | Include | IV |
| Çaylan et al. (2021) [19] | Analytical cross-sectional studies | 6 | Include | IV |
| Dirlik et al. (2015) [44] | Analytical cross-sectional studies | 6 | Include | IV |
| Dogan et al. (2010) [53] | Case series | 7 | Include | IV |
| Esiyok et al. (2006) [54] | Case series | 8 | Include | IV |
| Güzel et al. (2013) [20] | Case series | 9 | Include | IV |
| Hsieh et al. (2018) [55] | Prevalence Studies | 8 | Include | IV |
| Işik and Eşitti (2015) [56] | Qualitative studies | 7 | Include | No level of evidence |
| Işın et al. (2020) [7] | Analytical cross-sectional studies | 6 | Include | IV |
| Işın and Peden (2022) [10] | Prevalence Studies | 8 | Include | IV |
| Işın et al. (2021) [18] | Analytical cross-sectional studies | 6 | Include | IV |
| Ketenci et al. (2022) [57] | Case series | 7 | Include | IV |
| Koca et al. (2019) [58] | Case series | 7 | Include | IV |
| Lakadamyalı et al. (2008) [35] | Case series | 6 | Include | IV |
| Lapa et al. (2012) [33] | Analytical cross-sectional studies | 6 | Include | IV |
| Lin et al. (2015) [59] | Prevalence Studies | 8 | Include | IV |
| Mollaoğlu et al. (2013) [60] | Case series | 8 | Include | IV |
| Orhan (2020) [61] | Case Series | 6 | Include | IV |
| Petrucci et al. (2019) [62] | Prevalence Studies | 8 | Include | IV |
| Şık et al. (2022) [46] | Case Series | 8 | Include | IV |
| Şık et al. (2021) [22] | Case Series | 8 | Include | IV |
| Şimşek and Satar [63] (2013) | Case Series | 6 | Include | IV |
| Söyüncü et al. (2008) [36] | Case series | 7 | Include | IV |
| Ta**ş**kesen et al. (2015) [21] | Analytical cross-sectional studies | 5 | Include | IV |
| Tunçez et al. (2022) [64] | Analytical cross-sectional studies | 5 | Include | IV |
| Turgut (2012) [39] | Analytical cross-sectional studies | 6 | Include | IV |
| Turgut and Turgut (2012) [45] | Analytical cross-sectional studies | 6 | Include | IV |
| Turgut and Turgut (2014) [9] | Analytical cross-sectional studies | 5 | Include | IV |
| Turgut et al. (2016) [37] | Quasi-Experimental Studies | 8 | Include | III-2 |
| Uzun et al. (2009) [65] | Analytical cross-sectional studies | 5 | Include | IV |
| Yayci et al. (2011) [66] | Analytical cross-sectional studies | 4 | Include | IV |
| Balcı et al. (2018) [67] | Analytical cross-sectional studies | 5 | Include | IV |
| Yıldırım et al. (2020) [68] | Case series | 7 | Include | IV |
| Küçük et al. (2020) [69] | Case series | 7 | Include | IV |
| Cömert et al. (2014) [70] | Case series | 7 | Include | IV |
| Türkoğlu et al. (2014) [42] | Analytical cross-sectional studies | 6 | Include | IV |
| Beydili et al. (2016) [34] | Analytical cross-sectional studies | 6 | Include | IV |
| Demir et al. (2017) [71] | Analytical cross-sectional studies | 5 | Include | IV |
| Tutanç et al. (2011) [72] | Analytical cross-sectional studies | 5 | Include | IV |
| Tutanç et al. (2011) [73] | Case series | 7 | Include | IV |
| Arslan et al. (2004) [74] | Analytical cross-sectional studies | 5 | Include | IV |
